# Change in subjective well-being and the associated costs of a woman-targeted presbyopia correction programme among older craftswomen in Zanzibar: a cost-outcome and scenario analysis

**DOI:** 10.1136/bjo-2024-325887

**Published:** 2025-01-29

**Authors:** Ving Fai Chan, Omar Juma Othman, Ai Chee Yong, Christine Graham, Carlos Price-Sanchez, Bhagyalaxmi Shivalingam Pillai, Eleanor Holland, Emma McConnell, Jamison Jones, Adrianna Farmer, Michelle Fernandes Martins, Kajal Shah, Damaris Mulewa, Ronnie Graham, Eden Mashayo, Fatma Omar

**Affiliations:** 1 Queen's University Belfast, Belfast, UK; 2 University of KwaZulu-Natal, Durban, South Africa; 3 Zanzibar Ministry of Health, Zanzibar, Mkoa wa Unguja Mjini Magh, United Republic of Tanzania; 4 Loyola University Chicago, Chicago, Illinois, USA; 5 Dublin Institute of Technology, Dublin, Dublin, Ireland; 6 Independent Researcher, Nairobi, Kenya; 7 Independent Researcher, Scotland, UK; 8 Vision Care Foundation, Dar-es-Salaam, United Republic of Tanzania

**Keywords:** Public health, Epidemiology

## Abstract

**Background:**

A pilot eyecare programme aimed to address the urgent eye health needs of older Zanzibari craftswomen. We investigated the impact of correcting presbyopia on their subjective well-being (SWB) 6 months post-correction and assessed the cost-effectiveness of a women-targeted presbyopia correction programme.

**Methods:**

This study involved Zanzibari craftswomen aged 40 and older with presenting and corrected distance visual acuity better than 6/12 in both eyes and were presbyopic. Using a before–after method, we assessed SWB on a 10-point scale before and after providing free spectacles. Mean SWB scores and differences pre-correction and post-correction were calculated. Programme costs were analysed to determine the cost per SWB score gained and the monthly cost for each SWB score improvement. Additionally, scenario analysis estimated costs for 12 approaches.

**Results:**

Of 282 craftswomen, 209 met the eligibility criteria. SWB scores significantly increased from 3.34 to 8.14 post correction (p<0.001). Screening costs totalled US$12 885.84, with an average cost of US$45.69 per craftswoman screened and US$61.66 per presbyopia identification. The total programme cost was US$14,574.69. One hundred fifty-four craftswomen experienced increased SWB, with a total of 747 score gains. Achieving one SWB score improvement cost an average of US$19.50, with a monthly average cost of US$3.40 per improvement. Utilising woman screeners, organised transport and ready-made spectacles appeared to be the most cost-effective approach.

**Conclusion:**

Correcting presbyopia through a targeted eyecare programme significantly enhanced SWB among craftswomen. While the programme seems cost-effective, further research is warranted to explore long-term economic benefits and definitively assess cost-effectiveness in larger studies.

**WHAT IS ALREADY KNOWN ON THIS TOPIC**

- The prevalence of uncorrected presbyopia among older Zanzibari craftswomen was high, but spectacle coverage was low. Provision of presbyopia correction to craftswomen has shown an immediate significant improvement in subjective well-being by 2.67 points.

**WHAT THIS STUDY ADDS**

- This first study examined the relationship between presbyopia correction and subjective well-being among older woman entrepreneurs living in a Muslim-majority patriarchal society found that 6 months after the correction of presbyopia with spectacles significantly improves subjective well-being among craftswomen, and that a woman-targeted programme is cost-effective, with opportunities for further optimisation based on a comprehensive scenario analysis.

**HOW THIS STUDY MIGHT AFFECT RESEARCH, PRACTICE OR POLICY**

- The findings of this study could be used for planning and advocating for woman-targeted programmes and improving access to eye health-related services in low-income and middle-income countries to achieve the Sustainable Development Goals (SDGs), particularly SDG5, which aims to achieve gender equality and women’s empowerment.

## Background

 With over 1 billion people experiencing presbyopia worldwide,^1^ substantial evidence highlights the impact of corrective measures on vision function, quality of life and work productivity.^2^,^5^ These findings have led to a heightened global focus on the necessity for spectacle correction in adults over the past decades. Despite spectacle correction being a safe and affordable intervention for refractive errors, the global burden of vision impairment and blindness disproportionately impacts women.^6 7^ Women constitute 56% of the 36 million blind individuals and 55% of the 217 million with moderate to severe vision impairment.^8^ Consequently, women face diminished access to education, restricted employment opportunities, social exclusion and an elevated risk of violence compared with men.^6^ This disparity is exacerbated by factors such as the neglect of female health priorities, and that age-related eye diseases are more prevalent in women due to longer life expectancy.^7^

The economic impact of presbyopia (a condition affecting near vision), prevalent among individuals aged 40 and older, is particularly significant for women in low and middle-income countries (LMICs) where approximately 740 million are engaged in informal employment.^9^ Given the economic challenges faced by women in these countries, many are compelled to forgo retirement, as their households depend on their financial support.^10^ This underscores the substantial burden borne by women in addressing vision-related challenges amidst broader socioeconomic constraints. Ironically, literature from the last decade showed only three eye health programmes dedicated to women in LMICs^11^,^13^—none of which have been evaluated for cost-effectiveness.

Zanzibar is an illustrative case: despite women constituting around 51% of the population in 2019, with 23% acting as household heads supporting an average of nine unemployed individuals—outnumbering their male counterparts who typically supported four—44% of these female household leaders lacked formal education.^14^ This led many to resort to home-based crafts to generate income,^14^ heavily relying on near vision. Despite the importance of near vision to craftswomen, Muslim principles and patriarchal norms in Zanzibar paradoxically present barriers that impede women’s access to eye care. These barriers include lack of awareness, cultural constraints and financial obstacles that exacerbate the vision-related challenges faced by these women.^15^ In response, the Ministry of Health in Zanzibar initiated a targeted eyecare pilot programme in 2022 called Women’s Empowerment through Investing in Zanzibari Craftswomen’s Eyesight (WE-ZACE). This initiative aims to empower older Zanzibari craftswomen entrepreneurs by correcting their presbyopia, as their work is heavily dependent on precise near vision.^15 16^

WE-ZACE conducted several studies, including a cross-sectional study of 263 craftswomen, revealing that 86.6% had presbyopia, with only 0.99% adequately corrected.^17^ These findings were similar to the population’s presbyopia prevalence of 89.2%, but demonstrated significantly lower spectacle coverage compared with the population’s 17.7% in 2010.^18^ Additionally, interviews with 24 craftswomen with presbyopia revealed that they believed near-vision spectacle correction could improve their economic, psychological, political and educational empowerment.^19^ They also believed that spectacle correction could enhance their ability to buy things for themselves, increase their self-confidence, increase their participation in social activities as well as enable them to educate others and lead their communities.^19^

Our study employed subjective well-being (SWB) as a metric for assessing women’s empowerment and evaluating the impact of our interventions. SWB is a self-reported measure of well-being that encompasses an individual’s emotions, thoughts and satisfaction with various aspects of their life.^20^ A survey of 217 women entrepreneurs found that 87.6% rated their current SWB as suboptimal (10-point Cantril’s ladder score of less than 5).^21^ However, shortly after they were corrected (within 30 min to an hour), their mean SWB score improved significantly by 2.67.^21^ Hence, our subsequent question is *What is the longer-term effect of presbyopia correction on their SWB?*

Our literature review revealed only one study that assessed the cost-effectiveness of refractive error correction, including presbyopia, offered via a hospital in Zambia with a small sample of 44 patients. This study found that the incremental costs per quality-adjusted life years gained was US$375 for refractive error correction.^22^ As such, our study aimed to calculate the cost of intervention and the subsequent change in SWB score 6 months after correction of presbyopia. Then, a cost-outcome analysis was conducted to determine the programmatic resources consumed to achieve a change in SWB scores. Demonstrating that a woman-targeted eyecare programme could be cost-effective in addressing unmet need of presbyopia correction early on is crucial for effective resource management and accountability.

## Methods

The research was implemented among 19 women cooperatives on the two main islands of Zanzibar (Unguja and Pemba) from 16 February 2023 to 4 October 2023.

### Patient and public involvement

Craftswomen entrepreneurs and local stakeholders were involved in the development of the WE-ZACE programme through two patient and public involvement meetings during the initiation and development stage. They provided input on the research questions, suggestions on timing for recruitment and implementation. Craftswomen entrepreneurs and local stakeholders were invited to the dissemination of the findings, with a breakout session to obtain their views and suggestions.

### Programme description

#### Kick-off meeting and training

In October 2022, a project management team was formed to facilitate the implementation and evaluation of WE-ZACE. A kick-off meeting was held to set objectives, roles, responsibilities and communication plans. Four optometrists/ophthalmic clinical officers and eight data collectors underwent a 4-day training session in February 2023. The training aimed to standardise vision screening, prescribing guidelines as well as recording and referral protocols.

#### Screening, eye examination, management and referral

For the study, Craftswomen aged 40 years and older in Zanzibar underwent eye examinations by ophthalmic clinical officers/optometrists Distance visual acuity (VA) was assessed using Tumbling E Snellen charts at 6 m. Individuals with VA worse than 6/12 in either eye underwent further testing to exclude eye morbidities. Those with no eye morbidities were given a refractive error test if their distance vision was 6/12 or better or if they had a correctable refractive error. Individuals were considered presbyopic if near vision was worse than N8 at 40 cm. To be eligible, participants had to have good distance vision, be presbyopic and not own near-vision spectacles. Those with other non-refractive vision problems were excluded, and individuals requiring further management (eg, cataract, posterior eye diseases) were referred to Mnazi Mmoja Hospital in Unguja.

#### Measuring SWB scores

Our study employed SWB as a metric to assess women’s empowerment and evaluate the impact of our interventions. As mentioned, SWB is a self-reported measure that reflects an individual’s emotions, thoughts and satisfaction with various aspects of their life.^20^ We used Cantril’s ladder, a validated and widely used tool for assessing SWB in diverse populations, including low-resource settings.^23^ Cantril’s ladder is a 10-point scale where participants are asked to evaluate their current position on the ‘ladder of life’, with 10 representing the best possible SWB and 1 the worst possible SWB. This tool was selected for its simplicity, cross-cultural validity and its ability to capture individuals’ subjective perceptions of empowerment and overall welfare.

In our study, before receiving spectacles, eligible craftswomen rated their SWB using this scale. While Cantril’s ladder does not decompose SWB into specific domains (eg, health, financial status, social relationships), it provides a holistic perspective on participants’ sense of empowerment and well-being. Previous research has demonstrated its reliability and sensitivity in capturing changes in SWB over time,^24^ making it particularly suitable for our population and intervention. Six months later, the women were then asked to rate their SWB again after their eyesight was corrected. The mean SWB scores and mean SWB score difference before and after vision correction was calculated, and a paired sample t-test was employed to assess the significance of the change in SWB scores, with a significance level set at 5%. No multiple imputation was conducted due to a loss of follow-up exceeding 10%, which multiple imputation would have introduced bias.^25^ Data analysis was performed using the Statistical Package for the Social Sciences V.24. Full dataset is available at Zenodo dataset repository.^26^

#### Cost determination

Project account records were reviewed to collect information on resources used by the interventions (resource types, numbers and unit costs). Primary data collection was conducted by interviewing key informants such as representatives of the Ministry of Health (n=2), optometrists (n=4), project coordinator (n=1), project administrator (n=1) and principal investigator (n=1). Informed consent was obtained from key informants prior to their participation in the study. The cost categories with their cost components are shown in table 1. Costing was done for the period October 2022 to August 2023.

**Table 1:** Cost categories and cost components for the project

Cost category	Cost components
Meetings	Participants’ per diem, venue rental, refreshment for one project kick-off meeting, one multi-stakeholder planning meeting and two local project team meetings
Training of ophthalmic officers and optometrists	Participants’ per diem, venue rental, refreshment
Staff salaries	Full-time staff for 6 months
Screening kit	Occluder, VA chart, torch, measuring tape and vision cards.
Consumables	Spectacles
Outreach activities	Optometrist hourly rate X time spent on examining and prescribing spectacles*, Data collectors’ wages, transport†, accommodation, communication‡

* Optometrists’ time spent on examining and managing the women varied between 10 min and 30 min depending on the complexity of the case at hand.

† Means of transportation included car hire and fuel US$ 65.37 per car per day depending on distance from the cooperatives.

‡ Money spent on mobile phone communication was US$ 0.50 to US$ 0.90 per day.

VA, visual acuity.

#### Cost-outcome analysis

The cost and outcome matrix of the WE-ZACE programme is presented in online supplemental material 1, utilising a 10-point scale of Cantril’s score, where a score of 5 or below is considered suboptimal, and above 5 is considered optimal. The matrix consists of a 10×10 grid, with the rows representing SWB scores before correction (input) and the columns representing SWB scores 6 months after correction (output). The matrix mechanics are as follows:

- The women’s SWB was evaluated before correction and placed in the appropriate horizontal cell (eg, a prerating of 3 would place a woman in the third horizontal cell).
- The women’s SWB was reassessed 6 months after the spectacles were provided and placed in the appropriate vertical cell (eg, using the previous example, a postrating of 4 would place the women in the third and fourth matrix cell, indicating improvement; a postrating of 1 would place the women in the third and first matrix cell, indicating regression; a postrating of 3 would leave the women in the third matrix cell, indicating no change).

Improvement is indicated by horizontal movement to the right of the diagonal line, regression is indicated by horizontal movement to the left of the diagonal line, and no change is indicated by remaining on the diagonal line. The cost data are accumulated by first computing costs by units of services provided to women, aggregating these unit costs across each 10×10 cell and summing these programme costs at each score level. Then the average cost of service, the cost-ratio of those with suboptimal to optimal SWB at the beginning and end of the programme and the average cost per woman with improved SWB were calculated.

Given the pilot nature of this study, we conducted scenario analyses on the costs and cost-effectiveness of the programme across eight potential implementation methods. These methods varied based on the type of screeners (women screeners, ophthalmic personnel or mixed), management approach (outreach vs supported transport) and type of spectacles (custom-made vs ready-made). Assumptions included ophthalmic personnel salaries being three times higher than craftswomen, custom-made spectacles costing 3.2 times more than ready-made ones, craftswomen requiring 2 days of training and organised transport costing US$4 per person for a round trip.

Subsequently, we conducted a one-way sensitivity analysis to assess the impact of cost variations on the outcomes of the programme. Key cost variables, including screening costs, programme costs, cost per woman screened and cost per woman identified with presbyopia, were varied by±20% to simulate plausible changes in input assumptions. Results were analysed to identify the most sensitive parameters and visualised using tornado diagrams to illustrate the extent of variation across scenarios.

## Results

### Craftswomen screened and identified to have presbyopia

At the outset, 282 craftswomen were screened, with 209 identified as having uncorrected presbyopia and meeting the study’s eligibility criteria. At the end of the intervention period, only 157 craftswomen (75.1%) could be followed up. At the end of the study, there were significantly more craftswomen from Pemba than Unguja and more 40–50-year olds than older than 50-year olds than at the beginning of the study (table 2).

**Table 2:** Comparison of those included in baseline and those successfully followed up 6 months later

	Before correction	After correction (6 months)		P value
	Total screened n=209	Successfully follow-up (n=157, 75.1%)	Loss-to-follow-up (n=52, 24.9%)	
Locations				
Unguja	111 (53.1%)	75 (47.8%)	36 (69.2%)	p<0.001
Pemba	98 (46.9%)	82 (52.2%)	16 (30.8%)	
Age group (years)				
40–50	102 (48.8%)	79 (50.3%)	23 (44.2%)	p<0.001
>50 years old	107 (51.2%)	78 (49.7%)	29 (55.8%)	
Level of education				
No formal education	24 (11.6%)	18 (11.5%)	6 (11.8%)	p=0.373
Did not complete primary				
Education	26 (12.6%)	20 (12.8%)	6 (11.8%)	
Completed primary education	94 (45.4%)	66 (42.3%)	28 (54.9%)	
Completed secondary education	63 (30.4%)	52 (33.3%)	11 (21.6%)	
Missing=2				
Type of craftwork engaged				
Weaving	114 (54.5%)	81 (51.6%)	33 (63.5%)	p=0.156
Tailoring and sewing	66 (31.6%)	51 (32.5%)	15 (28.8%)	
Pottery	10 (4.8%)	7 (4.5%)	3 (5.8%)	
Producing oil and making soaps	19 (9.1%)	18 (11.5%)	1 (1.9%)	
Years working as craftswomen				
10 years and lesser	139 (66.8%)	106 (67.9%)	33 (63.5%)	p=0.078
>10 years	69 (33.2%)	50 (32.1%)	19 (36.5%)	
Missing=1				
Marital status				
Single	3 (1.4%)	2 (1.3%)	1 (1.9%)	p=0.685
Married	158 (75.6%)	117 (74.5%)	41 (78.8%)	
Widowed	27 (12.9%)	20 (12.7%)	7 (13.5%)	
Separated/divorced	21 (10.0%)	18 (11.5%)	3 (5.8%)	
Number of children				
None	4 (1.9%)	4 (2.5%)	0 (0.0%)	p=0.291
1–4	59 (28.2%)	47 (29.9%)	12 (23.1%)	
5 or more	146 (69.9%)	106 (67.5%)	40 (76.9%)	
Number of dependents				
None	13 (6.2%)	8 (5.1%)	5 (9.6%)	p=0.215
1–4	113 (54.1%)	82 (52.2%)	31 (59.6%)	
5 or more	83 (39.7%)	67 (42.7%)	16 (30.8%)	
Prescription of near vision spectacles				
+1.00D to+1.75D	58 (27.8%)	46 (29.3%)	12 (23.1%)	p=0.683
+2.00D to+2.75D	101 (48.3%)	74 (47.1%)	27 (51.9%)	
>+2.75D	50 (23.9%)	37 (23.6%)	13 (25.0%)	

### SWB of craftswomen before and 6 months after correction and the mean change in SWB

Before the correction, the mean SWB score was 3.34 (95% CI 3.06 to 3.62), while after correction, it increased significantly to 8.14 (95% CI 7.89 to 8.38), indicating a substantial rise of 4.80 points (95% CI 4.46 to 5.14; p<0.001). We noted significant increases in SWB scores across all demographic categories, with no significant differences observed within these categories (table 3).

**Table 3:** Mean subjective well-being scores before and 6 months after correction, and mean difference in subjective well-being scores

	Mean SWB score before correction (SD)	Mean SWB score 6 months after correction (SD)	Mean difference score (95% CI)	P value
Locations				
Unguja	3.72 (1.928)	8.07 (1.563)	4.35 (3.84, 4.82)	0.11
Pemba	2.99 (1.511)	8.21 (1.537)	5.22 (4.77, 5.69)	
Age group (years)				
40–50	3.47 (1.859)	8.09 (1.554)	4.62 (4.14, 5.09)	0.287
>50 year old	3.21 (1.646)	8.19 (1.546)	4.99 (4.57, 5.47)	
Level of education				
No formal education	3.17 (1.425)	8.33 (1.572)	5.17 (4.11, 6.13)	0.907
Did not complete primary education	3.00 (1.556)	7.80 (1.240)	4.80 (3.89, 5.65)	
Completed primary education	3.38 (1.726)	8.12 (1.603)	4.74 (4.26, 5.29)	
Completed secondary education	3.44 (1.984)	8.27 (1.573)	4.83 (4.25, 5.42)	
Missing=2				
Type of craftwork engaged				
Weaving	3.27 (1.696)	7.91 (1.575)	4.64 (4.21, 5.09)	0.607
Tailoring and sewing	3.45 (1.880)	8.33 (1.506)	4.88 (4.23, 5.56)	
Pottery	2.57 (0.976)	8.29 (1.704)	5.71 (4.20, 7.00)	
Producing oil and making soaps	3.61 (1.914)	8.56 (1.423)	4.94 (4.08, 5.92)	
Years working as craftswomen				
10 years and lesser	3.18 (1.584)	8.17 (1.636)	4.99 (4.59, 5.43)	0.099
>10 years	3.68 (2.065)	8.06 (1.361)	4.38 (3.82, 4.98)	
Missing=1				
Marital status				
Single	3.00 (1.414)	6.00 (1.414)	3.00 (3.00, 3.00)	0.05
Married	3.35 (1.783)	8.15 (1.546)	4.80 (4.42, 5.20)	
Widowed	3.90 (1.861)	7.95 (1.669)	4.05 (3.19, 5.08)	
Separated/divorced	2.67 (1.328)	8.50 (1.295)	5.83 (5.00, 6.71)	
Number of children				
None	2.50 (1.000)	9.00 (0.000)	6.50 (5.50, 7.00)	0.28
1 to 4	3.47 (2.145)	8.21 (1.654)	4.74 (4.00, 5.45)	
5 or more	3.31 (1.582)	8.08 (1.523)	4.76 (4.36, 5.18)	
Number of dependents				
None	2.13 (1.126)	8.50 (1.069)	6.38 (5.00, 7.80)	0.098
1 to 4	3.50 (1.887)	8.16 (1.621)	4.66 (4.19, 5.14)	
5 or more	3.28 (1.603)	8.07 (1.511)	4.79 (4.32, 5.24)	
Prescription of near vision spectacles				
+1.00D to+1.75D	3.63 (2.069)	7.98 (1.437)	4.35 (3.69, 4.95)	0.222
+2.00D to+2.75D	3.11 (1.601)	8.15 (1.576)	5.04 (4.55, 5.52)	
>+2.75D	3.43 (1.608)	8.32 (1.634)	4.89 (4.19, 5.65)	
Total	3.34 (1.76)	8.14 (1.55)	4.80 (4.46, 5.14)	<0.001

P value comparing proportion difference before and 6 months after correction.

SWB, subjective well-being.

### Cost outcome analysis

Out of the 282 women screened, 209 were identified as having presbyopia. The total cost of screening was US$12 885.84, with recurring costs totalling 84.7% (US$10 914.30) The screening cost per craftswoman was US$45.69, and the cost to identify one with presbyopia was US$61.66. The total programme cost was US$14 574.69 (additional spectacles expenses of US$1 688.85). Out of 157 women who were effectively followed up, the cost per craftswoman successfully followed up amounted to US$92.83 (table 4).

**Table 4:** Cost category and costs for the programme

Cost category	Cost (US$)	Once-off/recurring cost
Meetings	1736.83	Once-off
Training of ophthalmic officers and optometrists	109.33	Recurring
Staff salaries	5128.20	Recurring
Screening kit	246.02	Once-off
Outreach activities	5665.46	Recurring
Total screening cost	12 885.84	–
Cost per woman screened (n=282)	45.69	–
Cost per woman identified to have presbyopia (n=209)	61.66	–
Spectacles	1688.85	Recurring
Total programme cost	14 574.69	–
Cost per woman successfully followed-up (n=157) in 6 months	92.83	–
Cost to gain one SWB score per month in SWB (n=154, total score gained in 6 months=747	3.40	–
Cost to regress one SWB score per month in SWB (n=3, total score lost in 6 months=7)	7.07	–

SWB, subjective well-being.

There were 143 craftswomen with suboptimal SWB score and 14 with optimal SWB scores at the beginning of the programme, incurring US$13 275.04 and US$1 518.28, respectively. At the end of the programme, 154 craftswomen had an improvement in their SWB, with a total of 747 score gains. The average cost to achieve one SWB score improvement was US$19.50, with an average monthly cost of US$3.40 per one SWB score improvement among craftswomen. Three craftswomen had a regression in their SWB, with a total of 7 SWB scores regression. The average cost per month of regression by one SWB score among the craftswomen was US$7.07. Detailed calculation is found in online supplemental material 2.

### Scenario analysis

Table 5 shows the costs for 12 combinations of approaches to deliver women-targeted presbyopia correction programme. Screening costs are most influenced by the type of screeners and management methods used. The lowest screening cost is projected with the use of woman screeners and paid organised transport to primary care clinics for examination (US$5327.28), while the programme cost is minimised when ready-made spectacles are combined with woman screeners and paid organised transport (US$5855.04). With this combination of using woman screeners, paid organised transport and ready-made spectacles, the costs per woman screened and per woman identified with presbyopia are US$18.89 and US$25.49, respectively. The second most cost-efficient screening option is utilising a mix of woman screeners and ophthalmic personnel and paid organised transport to primary care clinics for examination (US$6181.88), and programme costs could be minimised when ready-made spectacles are used (US$6709.64).

**Table 5:** Scenario analysis on costs for 12 combinations of approaches to deliver women-targeted presbyopia correction programme

Scenarios	A	B	C	D	E	F	G	H	I	J	K	L
Screening costs (US$)	5327.28	9576.37	9576.37	5327.28	8636.75	8636.75	12 885.84	12 885.84	6181.88	6181.88	10 430.97	10 430.97
Cost per woman screened (US$)	18.89	33.96	33.96	18.89	30.63	30.63	45.69	45.69	21.92	21.92	36.99	36.99
Cost per woman identified to have presbyopia (US$)	25.49	45.82	45.82	25.49	41.32	41.32	61.66	61.66	29.58	29.58	49.91	49.91
Programme costs (US$)	5855.04	10 104.14	11 265.22	7016.13	9164.51	10 325.60	13 413.61	14 574.69	6709.64	7870.73	10 958.74	12 119.82

A, woman screener + supported transport + ready-made; B, woman screener + outreach + ready-made; C, woman screener + outreach + custom-made; D, woman screener + supported transport + custom-made; E, optometrist + supported transport + ready-made; F, optometrist + supported transport + custom=made; G, optometrist + outreach + ready-made; H, optometrist + outreach + custom-made; I, mix of woman screeners and ophthalmic personnel + supported transport + ready-made; J, mix of woman screeners and ophthalmic personnel + supported transport + custom-made; K, mix of woman screeners and ophthalmic personnel + outreach + ready-made; L, mix of woman screeners and ophthalmic personnel + outreach + custom-made .

The sensitivity analysis revealed that screening costs were the most influential factor, with±20% variations causing significant shifts in the cost per woman screened and identified with presbyopia across all scenarios. Programme costs also showed notable sensitivity, particularly in scenarios utilising optometrists and outreach methods, which had higher baseline costs. Scenarios involving woman screeners, ready-made spectacles and supported transport (eg, scenario A) demonstrated greater stability, with smaller absolute variations compared with mixed or optometrist-led approaches. Online supplemental material 3 shows the impact of cost variations on key metrics in the programme.

## Discussions

This study investigated the impact of presbyopia correction on SWB among elderly craftswomen and assessed the programme’s cost-effectiveness. We found that a woman-targeted presbyopia correction programme significantly improved the SWB of craftswomen, irrespective of their background. The programme identified 209 women with presbyopia at a screening cost of US$45.69 per woman, and an average monthly cost of US$3.40 to improve a craftswoman’s well-being score by one point per month. Utilising woman screeners, organised transport and ready-made spectacles appeared to be the most cost-effective approach.

Our findings revealed a statistically significant enhancement in SWB scores following presbyopia correction in craftswomen, with an average increase of 4.8 points. This improvement aligns with the existing literature indicating a positive correlation between corrected presbyopia and improved quality of life.^27^ Notably, the observed improvement surpassed the average SWB gains reported for other prevalent health interventions for older women (eg, exercise: 2.1 points,^28^ hormone replacement therapy: 1.7 points,^29^ cognitive behavioural therapy: 3.7 points).^30^ It is crucial to acknowledge that these studies used different research designs, and that individual responses to interventions may vary. The substantial impact of presbyopia correction on SWB might be attributed to its ability to enhance a woman’s capacity to perform daily tasks requiring near-vision (reading, sewing, using electronic devices).^19^ This can lead to increased feelings of independence, competence and overall well-being.^19^ Additionally, the study observed consistent improvement across all demographic categories, suggesting a broad positive impact of presbyopia correction. However, limitations may include insufficient sample size to detect subtle variations or unaccounted factors influencing SWB.

This study offers novel insights into the cost-effectiveness of a presbyopia correction programme for craftswomen. Screening all participants incurred a cost of approximately US$45 per person, increasing to just US$62 on identifying presbyopia due to a high presbyopia prevalence. A significant portion (84.7%) of these costs is recurring, presenting opportunities for optimisation. One potential strategy involves training craftswomen to replace ophthalmic personnel as champions for eye health screening. This could lead to a substantial cost reduction due to the lower daily wage of craftswomen compared with ophthalmic personnel (three times lower). Furthermore, an outreach-based service delivery model might be unsustainable^31^ and foster reliance on free services^32^ offered at cooperatives. We propose a long-term solution involving women-led organised transportation to nearby facilities for eye examinations and spectacles prescription at primary health clinics given its potential feasibility shown by Lavier’s *et al*’s study in 2011—79% were willing to take part in such approach.^33^ While the total programme cost approached US$15 000 to cover a cohort of 209 craftswomen, the cost per craftswoman with improved SWB was approximately US$93. This suggests potential cost-effectiveness, particularly considering the future use of ready-made spectacles^34^ (US$3.24/pair) that costs 3.2 times lower than custom-made spectacles (US$10.36/pair) used in this study, long-term benefits on SWB and the potential benefits to individual productivity.^3 4 35^

However, further economic analysis is necessary to explore potential long-term cost savings. Future research quantifying potential reductions in healthcare utilisation or absenteeism due to improved SWB would strengthen the economic case for the woman-targeted programme. Additionally, cost-effectiveness might differ with larger programmes, where economies of scale could reduce screening costs, but programme management might become more expensive.

This study provides valuable data for a cost-outcome analysis of the presbyopia correction programme. The initial cost for addressing suboptimal well-being (around US$13,275) serves as a baseline for comparison. With an average SWB gain of one unit for every US$20.40 spent and an average monthly cost to gain one SWB score is about US$3.40, which is half the cost compared with those experiencing SWB regression after correction, these underscores the programme’s potential efficiency. However, a more definitive judgement regarding the USD20.40 per unit SWB improvement trade-off necessitates considering long-term benefits (reduced healthcare use, increased productivity) and the distribution of these improvements among participants in a longer follow-up study.

Scenario and sensitivity analyses highlight the importance of optimising screeners and programme management for cost-effective eye health programmes. Employing female screeners, organised transportation and ready-made spectacles significantly reduce costs compared with current methods. Prioritising strategies that minimise screening costs and favour ready-made spectacles enhances cost-effectiveness and scalability, particularly in stable scenarios with low-cost methods and supported transport. Outreach methods involving optometrists or custom-made spectacles require contingency plans due to their higher sensitivity to cost changes. Although women screeners offer a cost-efficient alternative, their accuracy may depend on the quality and extent of their training as well as access to adequate screening tools. Future studies should evaluate whether additional training or supervision could optimise their performance while retaining cost advantages. Furthermore, more implementation research is required to fully understand the costs and benefits associated with different approaches.

In the Zanzibar setting, there are currently no community-based eye health interventions, which limits direct comparisons or integration of such models. However, our findings may serve as a foundation for developing community-based approaches tailored to the local context. Such interventions could expand accessibility by decentralising care and engaging local communities in screening and follow-up processes. Importantly, the absence of the existing community-based programmes emphasises the need for innovation in delivering eye care services in Zanzibar and similar settings. These considerations underscore the importance of future research to evaluate whether hybrid or decentralised models can enhance the scalability and sustainability of eye health programmes while addressing cultural and socioeconomic factors unique to these environments.

The study acknowledges limitations that warrant consideration, particularly the follow-up rate of 157 out of 209 eligible craftswomen after 6 months, which may affect the generalisability of the findings. Factors contributing to this loss could include logistical challenges like transportation difficulties, competing responsibilities such as work or family obligations and limited access to follow-up locations. Some participants may have perceived follow-up as unnecessary due to satisfaction with their initial experience or minimal perceived vision improvement. This loss to follow-up could introduce bias in the SWB results, as those who did not return may have had systematically different outcomes. For example, women who derived no benefit from the intervention might be underrepresented, potentially inflating SWB outcomes, while those with substantial improvements might have skipped follow-up, leading to underestimation of the intervention’s full impact. Notably, the programme identified and treated craftswomen with other medical conditions, such as distance refractive errors and cataracts, which, if included in the analysis, could have resulted in an over or underestimation of the actual cost-effectiveness of the entire initiative. Furthermore, it is imperative to recognise that the cost-outcome analysis provided is preliminary and did not consider potential fluctuations in programme management costs associated with large-scale implementations.

## Conclusion

This study demonstrates that presbyopia correction in older craftswomen is associated with a significant improvement in SWB, exceeding the effects observed with other common interventions. Furthermore, the pilot programme appears to be cost-effective. However, further research is needed to explore the programme’s long-term economic benefits and to definitively assess cost-effectiveness in larger implementations considering economies of scale and potential changes in programme management costs.

## Supplementary material

10.1136/bjo-2024-325887 online supplemental file 1

## Data Availability

Data are available in a public, open access repository.
