# Supplementary material for: Change in subjective well-being and the associated costs of a woman-targeted presbyopia correction programme among older craftswomen in Zanzibar: a cost-outcome and scenario analysis
Source: Br J Ophthalmol. 2025 Jan 29;109(8):e325887. doi: 10.1136/bjo-2024-325887 (PMC12320605; doi:10.1136/bjo-2024-325887)
Supplement: online supplemental file 1 [file bjo-109-8-s001.pdf]

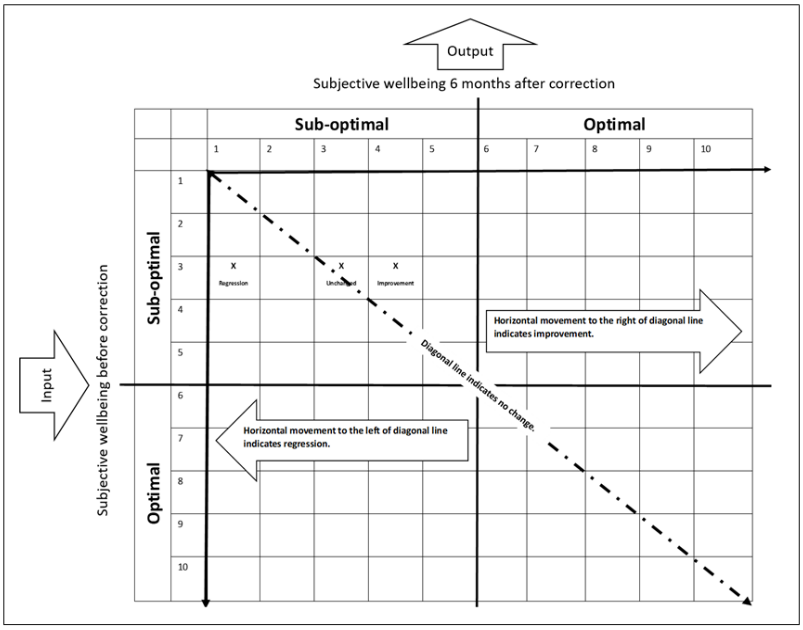

Supplemental material 1: Cost and outcome matrix

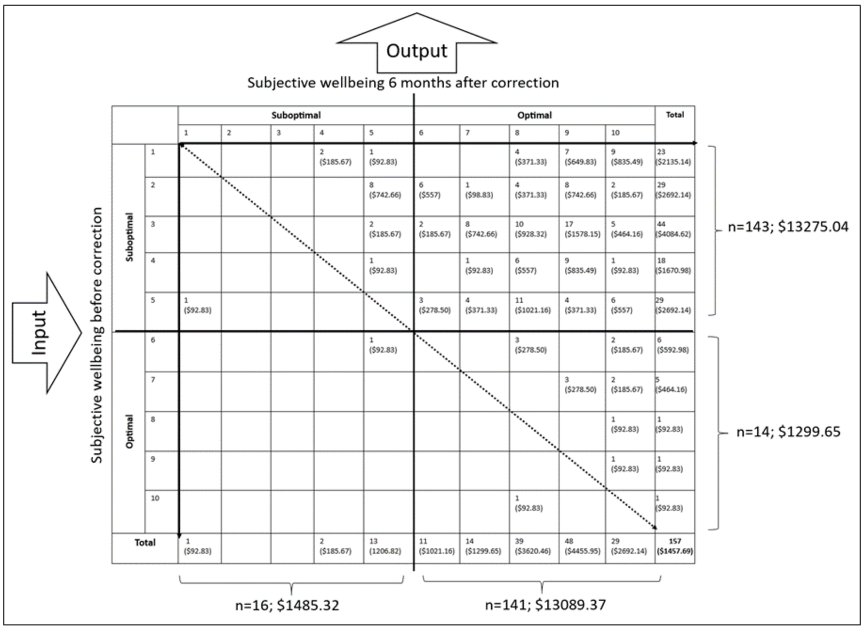

		Suboptimal					Optimal					Total
		1	2	3	4	5	6	7	8	9	10	
Suboptimal	1				2 ($185.67)	1 ($92.83)			4 ($371.33)	7 ($649.83)	9 ($835.49)	23 ($2135.14)
	2					8 ($742.66)	6 ($557)	1 ($98.83)	4 ($371.33)	8 ($742.66)	2 ($185.67)	29 ($2692.14)
	3					2 ($185.67)	2 ($185.67)	8 ($742.66)	10 ($928.32)	17 ($1578.15)	5 ($464.16)	44 ($4084.62)
	4					1 ($92.83)		1 ($92.83)	6 ($557)	9 ($835.49)	1 ($92.83)	18 ($1670.98)
	5	1 ($92.83)					3 ($278.50)	4 ($371.33)	11 ($1021.16)	4 ($371.33)	6 ($557)	29 ($2692.14)
Optimal	6					1 ($92.83)			3 ($278.50)		2 ($185.67)	6 ($592.98)
	7									3 ($278.50)	2 ($185.67)	5 ($464.16)
	8										1 ($92.83)	1 ($92.83)
	9										1 ($92.83)	1 ($92.83)
	10								1 ($92.83)			1 ($92.83)
Total		1 ($92.83)			2 ($185.67)	13 (1206.82)	11 ($1021.16)	14 ($1299.65)	39 ($3620.46)	48 ($4455.95)	29 ($2692.14)	**157 ($1457.69)**

Supplemental material 2: Cost and outcome matrix before and 6 months after correction

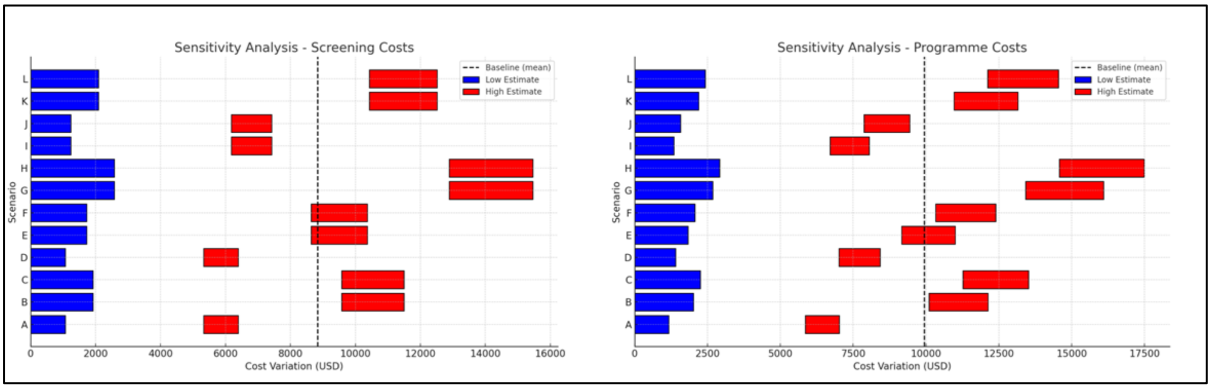

Supplemental material 3: Cost variations on key metrics in the programme – sensitivity analysis
